# Supplementary material for: Strength of carbon nanotubes depends on their chemical structures
Source: Nat Commun. 2019 Jul 10;10:3040. doi: 10.1038/s41467-019-10959-7 (PMC6620359; doi:10.1038/s41467-019-10959-7)
Supplement: Supplementary file 1 — Supplementary Information [file 41467_2019_10959_MOESM1_ESM.pdf]

SUPPLEMENTARY INFORMATION

Strength of carbon nanotubes depends on their chemical structures

Takakura *et al*.

Supplementary Note 1: Derivation of *f*(*θ*)

f(*θ*) in Eq. (1) of the main text is derived from the coordinate transformation of the stress tensor. A stress in a two-dimensional plane is described as a second-order tensor of the form:

$$\varepsilon = \begin{pmatrix} \varepsilon_{11} & \varepsilon_{12} \\ \varepsilon_{21} & \varepsilon_{22} \end{pmatrix}. \tag{1}$$

If the coordination system is rotated by an angle θ, the stress tensor in the new coordination system is related to the original one as:

$$\varepsilon' = \begin{pmatrix} \varepsilon_{11}' & \varepsilon_{12}' \\ \varepsilon_{21}' & \varepsilon_{22}' \end{pmatrix} = \begin{pmatrix} \cos\theta & \sin\theta \\ -\sin\theta & \cos\theta \end{pmatrix} \begin{pmatrix} \varepsilon_{11} & \varepsilon_{12} \\ \varepsilon_{21} & \varepsilon_{22} \end{pmatrix} \begin{pmatrix} \cos\theta & -\sin\theta \\ \sin\theta & \cos\theta \end{pmatrix}. \tag{2}$$

In our discussion, the *x*-axis in the original coordination system (*x*-*y*) and the *x*′-axis in the new one (*x*′-*y*′) are parallel to the tube axis and C-C bond direction, respectively. Therefore, under the uniaxial strain condition along the nanotube axis ($\varepsilon_{12} = \varepsilon_{21} = 0$), ε_{11}', the stress along the C-C bond direction, is given as

$$\begin{aligned} \varepsilon_{11}' &= \varepsilon_{11}\cos^2\theta - \nu\varepsilon_{11}\sin^2\theta \\ &= \frac{\varepsilon_{11}}{2}[(1-\nu) + (1+\nu)\cos 2\theta] \\ &= \varepsilon_{11} f(\theta), \end{aligned} \tag{3}$$

where ν is the Poisson ratio and $\varepsilon_{22} = -\nu\varepsilon_{11}$.

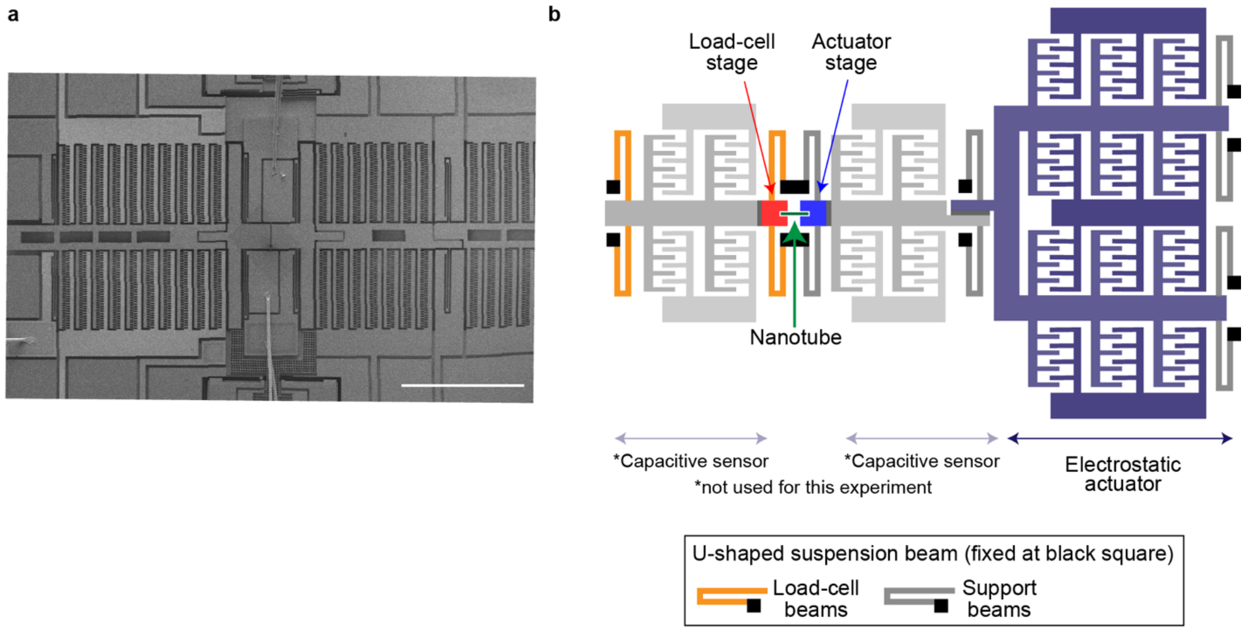

Supplementary Fig. 1 The microelectromechanical system (MEMS) tensile testing device for nanotubes. **a** Scanning electron microscope (SEM) image. Scale bar, 1 mm. **b** Schematic of the MEMS device.

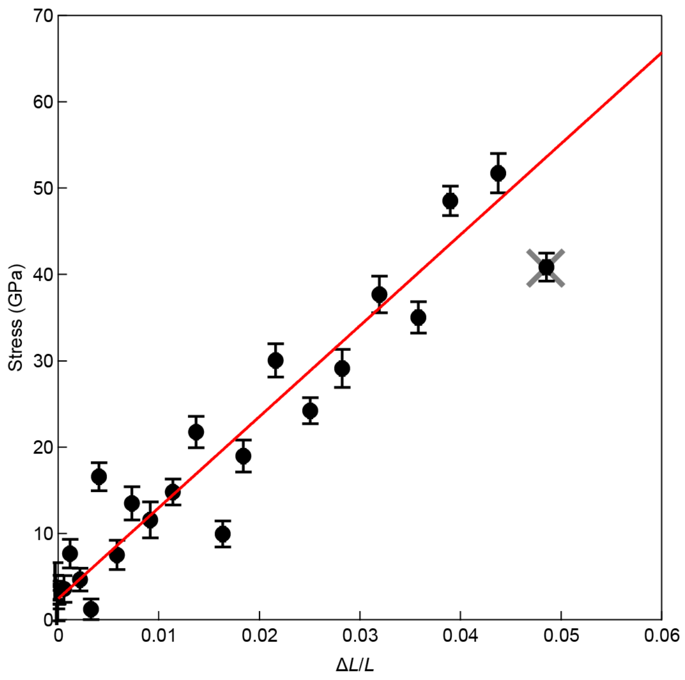

Supplementary Fig. 2 Stress–strain diagram. The chiral indices are (15,8). *L* is the initial distance between the two stages, and Δ*L* is the displacement, such that Δ*L*/*L* is equal to the nanotube strain when the nanotube does not slip on the stages. The red line is the linear fit to the data, yielding a Young's modulus of 1.05 ± 0.08 TPa, assuming no slip at the nanotube-stage interfaces. The error bars indicate the 95% confidence levels.

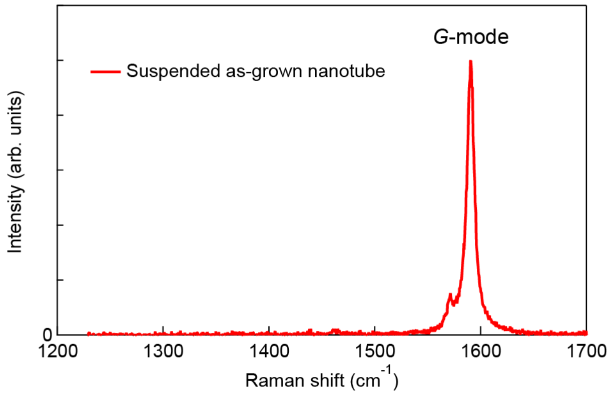

Supplementary Fig. 3 Example Raman spectrum of an individual suspended nanotube. The chiral indices are (18,8).

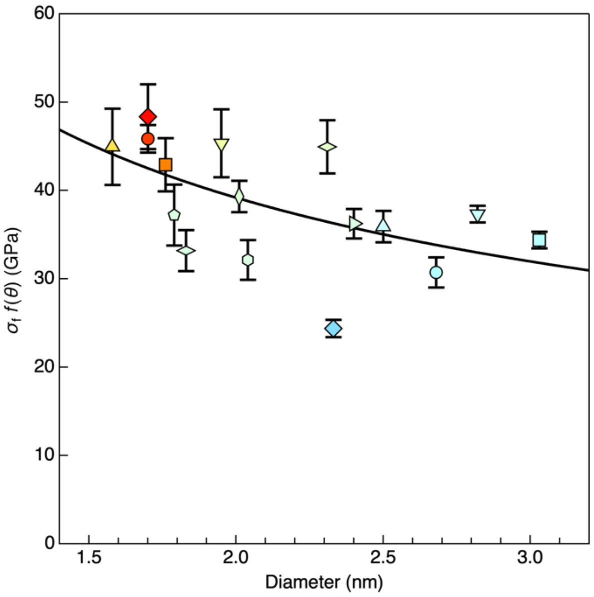

Supplementary Fig. 4 Empirical scaling factor for the diameter dependence of the tensile strength. The vertical axis is the product of the tensile strength, σ_{f}, and $f(\theta)$, where θ is the chiral angle and $f(\theta)$ is given by $(1/2)[(1-\nu)+(1+\nu)\cos 2\theta]$ ($\nu =$ 0.16 is the Poisson ratio of graphite). The solid curve is the best fit to the data, given as $\sigma_{\mathrm{f}} f(\theta) \propto d^{-\alpha}$, where $\alpha =$ 0.5 ± 0.2. The error bars indicate the 95% confidence levels.

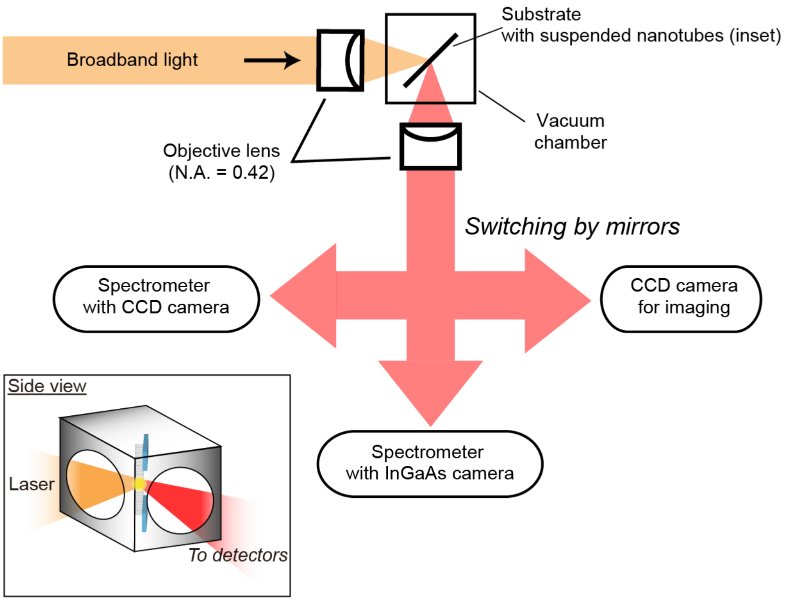

Supplementary Fig. 5 Schematic of the broadband Rayleigh spectroscopy of individual suspended nanotubes. Switching the mirrors changes the detection of the optical paths to either the charge-coupled device (CCD) camera for imaging, the CCD camera (1.2–2.8 eV) for spectroscopy, or the indium-gallium-arsenide (InGaAs) camera (0.8–1.4 eV) for spectroscopy. The inset shows the schematic side view of the vacuum chamber.

Supplementary Table 1 Summary of the observed exciton energies.

(n,m)	Exciton energies (eV)			
(13,12)	S_{22} 1.11	S_{33} 2.11	S_{44} 2.52	
(14,11)*	M_{11} 1.58, 1.62			
(14,12)	S_{22} 1.07	S_{33} 2.07	S_{44} 2.41	
(15,8)	S_{22} 1.20	S_{33} 2.14	S_{44} 2.76	
(15,12)	M_{11} 1.47, 1.50	M_{22} 2.66		
(16,14)	S_{22} 0.94	S_{33} 1.82	S_{44} 2.16	
(17,9)	S_{22} 1.03	S_{33} 2.08	S_{44} 2.30	
(20,9)	S_{22} 0.92	S_{33} 1.88	S_{44} 2.09	
(22,5)	S_{22} 0.95	S_{33} 1.96	S_{44} 2.12	
(22,13)	M_{11} 1.16~1.19	M_{22} 2.16, 2.30		
(25,11)*	S_{33} 1.57	S_{44} 1.78	S_{55} 2.56	
(25,14)*	S_{33} 1.46	S_{44} 1.70	S_{55} 2.40	S_{66} 2.50
(27,5)	S_{22} 0.85	S_{33} 1.54	S_{44} 2.05	S_{55} 2.42
(29,1)	S_{22} 0.87	S_{33} 1.56	S_{44} 2.11	S_{55} 2.41
(30,10)*	S_{33} 1.39	S_{44} 1.60	S_{55} 2.33	
(32,11)	M_{11} 0.95, 0.97	M_{22} 1.77, 1.92	M_{33} 2.47	

S_{ii} and M_{ii} indicate the ith subband excitons of the semiconducting and metallic nanotubes, respectively. The nanotubes indicated by asterisks exhibited no excitonic resonance in the 0.8–1.2 eV photon energy range. The chiral indices (n, m) in the first column are assigned according to Ref. 1.

Supplementary Table 2. Tensile strength summary (experimental).

(*n*, *m*)	Diameter (nm)	Chiral angle (º)	Tensile strength (GPa)
(13,12)	1.70	28.7	66 ± 5
(14,11)	1.70	26.0	59 ± 2
(14,12)	1.76	27.5	57 ± 4
(15,8)	1.58	20.0	52 ± 5
(22,5)	1.95	10.0	47 ± 4
(29,1)	2.31	1.7	45 ± 3
(20,9)	2.01	17.6	44 ± 2
(17,9)	1.79	19.9	43 ± 4
(15,12)	1.83	26.3	43 ± 3
(16,14)	2.04	27.8	43 ± 3
(22,13)	2.40	21.6	43 ± 2
(25,11)	2.50	17.3	40 ± 2
(30,10)	2.82	13.9	40 ± 1
(32,11)	3.03	14.3	37 ± 1
(25,14)	2.68	20.8	36 ± 2
(27,5)	2.33	8.4	25 ± 1

Supplementary Table 3 Estimated tensile strength (strongest 100 nanotubes for d > 0.6 nm).

Rank	(*n*,*m*)	Diameter (nm)	Chiral angle (º)	Tensile strength (GPa)
1	(5,5)	0.68	30	94
2	(5,4)	0.61	26.3	91
3	(6,6)	0.81	30	86
4	(6,5)	0.75	27.0	83
5	(6,4)	0.68	23.4	82
6	(6,3)	0.62	19.1	80
7	(7,7)	0.95	30	79
8	(7,6)	0.88	27.5	78
9	(7,5)	0.82	24.5	76
10	(7,4)	0.76	21.1	74
11	(8,8)	1.09	30	74
12	(7,2)	0.64	12.2	73
13	(7,3)	0.70	17.0	73
14	(8,7)	1.02	27.8	73
15	(8,6)	0.95	25.3	72
16	(8,5)	0.89	22.4	70
17	(9,9)	1.22	30	70
18	(8,0)	0.63	0	69
19	(8,4)	0.83	19.1	69
20	(9,8)	1.15	28.1	69
21	(8,1)	0.67	5.8	68
22	(8,2)	0.72	10.9	68
23	(8,3)	0.77	15.3	68
24	(9,7)	1.09	25.9	68

25	(9,6)	1.02	23.4	67
26	(9,0)	0.70	0	66
27	(9,5)	0.96	20.6	66
28	(10,9)	1.29	28.3	66
29	(10,10)	1.36	30	66
30	(9,4)	0.90	17.5	65
31	(9,1)	0.75	5.2	64
32	(9,2)	0.79	9.8	64
33	(9,3)	0.85	13.9	64
34	(10,8)	1.22	26.3	64
35	(10,7)	1.16	24.2	63
36	(11,10)	1.42	28.4	63
37	(11,11)	1.49	30	63
38	(10,0)	0.78	0	62
39	(10,5)	1.04	19.1	62
40	(10,6)	1.10	21.8	62
41	(11,9)	1.36	26.7	62
42	(10,1)	0.82	4.7	61
43	(10,2)	0.87	8.9	61
44	(10,3)	0.92	12.7	61
45	(10,4)	0.98	16.1	61
46	(11,8)	1.29	24.8	61
47	(12,12)	1.63	30	61
48	(11,7)	1.23	22.7	60
49	(12,11)	1.56	28.6	60
50	(11,0)	0.86	0	59

51	(11,5)	1.11	17.8	59
52	(11,6)	1.17	20.4	59
53	(12,10)	1.49	27.0	59
54	(11,1)	0.90	4.3	58
55	(11,2)	0.95	8.2	58
56	(11,3)	1.00	11.7	58
57	(11,4)	1.05	14.9	58
58	(12,8)	1.37	23.4	58
59	(12,9)	1.43	25.3	58
60	(13,12)	1.70	28.7	58
61	(13,13)	1.76	30	58
62	(12,0)	0.94	0	57
63	(12,7)	1.30	21.4	57
64	(13,11)	1.63	27.2	57
65	(12,1)	0.98	4.0	56
66	(12,5)	1.18	16.6	56
67	(12,6)	1.24	19.1	56
68	(13,9)	1.50	24.0	56
69	(13,10)	1.56	25.7	56
70	(14,13)	1.83	28.8	56
71	(14,14)	1.90	30	56
72	(12,2)	1.03	7.6	55
73	(12,3)	1.08	10.9	55
74	(12,4)	1.13	13.9	55
75	(13,8)	1.44	22.2	55
76	(14,12)	1.76	27.5	55

77	(13,0)	1.02	0	54
78	(13,1)	1.06	3.7	54
79	(13,6)	1.32	18.0	54
80	(13,7)	1.38	20.2	54
81	(14,10)	1.64	24.5	54
82	(14,11)	1.70	26.0	54
83	(15,14)	1.97	28.9	54
84	(15,15)	2.03	30	54
85	(13,2)	1.10	7.1	53
86	(13,3)	1.15	10.2	53
87	(13,4)	1.21	13.0	53
88	(13,5)	1.26	15.6	53
89	(14,8)	1.51	21.1	53
90	(14,9)	1.57	22.8	53
91	(15,12)	1.83	26.3	53
92	(15,13)	1.90	27.6	53
93	(16,16)	2.17	30	53
94	(14,0)	1.10	0	52
95	(14,1)	1.14	3.4	52
96	(14,6)	1.39	17.0	52
97	(14,7)	1.45	19.1	52
98	(15,11)	1.77	24.9	52
99	(16,14)	2.04	27.8	52
100	(16,15)	2.10	28.9	52

References

1. Liu, K. *et al.* An atlas of carbon nanotube optical transitions. *Nat. Nanotech.* **7,** 325–329 (2012).
